# MM-SleepNet: An Automatic Sleep Staging Network Based on Modal Re-Weighting and Mamba

**DOI:** 10.3390/s26216872

**Published:** 2026-10-30

**Authors:** Bin Li, Aolin Lin, Yonghan Zhang, Zhuang Li

**Affiliations:** 1 School of Computer Science, Northeast Electric Power University, Jilin 132012, China; 2 School of Mathematical and Computer Sciences, Heriot-Watt University, Edinburgh EH14 4AS, UK

**Keywords:** sleep staging, PSG recordings, deep learning, modal re-weighting, Mamba

## Abstract

Sleep staging is the key step in sleep quality assessment and sleep disorder diagnosis. In clinical practice, doctors usually use polysomnography (PSG) to stage sleep. PSG contains physiological signals such as electroencephalography (EEG) and electrooculography (EOG) and is divided into continuous epochs of fixed duration. In order to achieve more efficient and accurate automatic interpretation of PSG, this paper proposes MM-SleepNet, an automatic sleep staging network based on modal re-weighting and Mamba. MM-SleepNet divides each epoch into several overlapping patches and uses a newly designed patch feature extractor to extract the features of each patch, in order to solve the problem of key waveforms in the patch being obscured by non-key waveforms throughout the epoch. Afterward, MM-SleepNet calculates the importance of different signals on patches at the same time point through a dynamic re-weighting module. MM-SleepNet uses a bidirectional Mamba encoder that considers positional encoding to learn contextual information across epochs to adapt to the transition patterns between sleep stages. The experimental results on the Sleep-EDF and ISRUC datasets show that the proposed MM-SleepNet outperforms state-of-the-art methods. In addition, ablation experiments have demonstrated the effectiveness of various modules in MM-SleepNet.

## 1. Introduction

Sleep is not merely ‘rest’; it is an essential physiological process for body repair, brain organization, and immune regulation. Sleep staging is a fundamental method for describing the state of overnight sleep, with the aim of dividing the continuous sleep process into several stages based on sleep depth. In clinical practice, sleep staging is usually based on polysomnography (PSG). PSG consists of various physiological signals such as electroencephalography (EEG) and electrooculography (EOG) and is the standard recording method for sleep signals. According to AASM sleep staging rules [1], overnight PSG recordings are segmented into 30 s sleep epochs. The AASM standard divides sleep stages into five categories based on sleep depth from shallow to deep: Wake, N1, N2, N3, and REM. Manually interpreting overnight sleep records is time-consuming and labor-intensive, whereas automatic sleep staging can improve sleep-monitoring efficiency and the diagnosis of sleep disorders [2].

Early research on automatic sleep staging often used a pattern recognition framework that combines manual features with traditional machine learning classifiers. For example, Fraiwan et al. [3] converted single-channel EEG into Choi–Williams distribution signals, continuous wavelet signals, and Hilbert–Huang transform signals, extracted Rényi entropy features, and finally used a random forest classifier to complete sleep staging. Alickovic and Subasi first denoise the single-channel Pz-Oz EEG, then extract statistical features of different frequency bands through discrete wavelet transform, and finally use an ensemble support vector machine to complete sleep stage classification [4]. Traditional machine learning methods heavily rely on manually designed features, and model performance is greatly affected by feature quality and expert experience, resulting in limited generalization ability. With the development of deep learning, researchers have begun to use neural networks to automatically learn discriminative features from raw physiological signals.

Supratak et al. [5] proposed DeepSleepNet, which uses dual-branch CNNs with different kernel lengths to extract features from a complete epoch. Eldele et al. [6] proposed AttnSleep, which enhances discriminative information through feature recalibration on the dual-branch convolutional features of the complete epoch. Lee et al. [7] proposed SleePyCo, which extracts deep convolutional features from a complete epoch and enhances the distinguishability of features from different sleep stages through contrastive learning. Wan et al. [8] proposed MA-CNN-BiLSTM, which extracts handcrafted features, including energy and entropy, from raw EEG and wavelet-decomposed rhythms, and combines CNN, BiLSTM, and SEAM for classification. Wang et al. [9] proposed CareSleepNet, which performs continuous convolution transformer encoding on epochs to aggregate global information into epoch-level features. Zhang et al. [10] proposed TSEDSleepNet, which extracts features from epochs using an EEG/EOG dual-stream encoder–decoder and performs multi-scale feature fusion on deep features. Huang et al. [11] proposed EEGCMCNet, which uses a multi-scale CNN to extract epoch features and combines an SE module with a capsule network for classification based on deep features. These methods typically use multi-layer convolution for feature extraction on complete epoch segments, accompanied by multiple downsampling operations. However, key waveforms that occur only locally, such as sleep spindles, K-complexes, and rapid eye movements, are prone to mixing with background waveforms during repeated convolution, weakening their distinguishability in deep features. At the same time, pooling and downsampling operations continuously compress the time dimension, which may weaken or even lose the position, duration, and feature details of key waveforms. The relevant methods and their limitations in key waveform representation are summarized in Table 1.

Due to the complementary role of different physiological signals such as EEG and EOG in sleep staging tasks, sleep staging methods based on multi-modal PSG signals have gradually become the mainstream for automatic sleep staging. Chambon et al. [12] introduced multi-modal physiological signals into an independent branch convolutional network and fused multi-modal information by concatenating multiple channel features on the same epoch. Jia et al. [13] proposed SalientSleepNet, which adds and fuses significant waveform features of different modalities in the same epoch. Zheng et al. [14] proposed MMASleepNet, which uses an attention mechanism to learn inter-modal dependencies on deep convolutional features of multi-modal independent branches in the same epoch. Hu et al. [15] proposed SingleSleep, which extracts features using a 0.5 s non-overlapping window within an epoch and models them using a Transformer. Its multi-modal extension, SingleSleepPlus, integrates epoch features of various modalities through cross-modal attention fusion. Dai et al. [16] proposed MultiChannelSleepNet, which extracts the frequency features of multi-modal physiological signals separately, concatenates them, and further learns multi-modal features through a Transformer encoder. In addition, Ma et al. [17] proposed ST-USleepNet, which extracts spatiotemporal features from multi-modal sleep signals through space-time maps. Liu and Zhang [18] proposed STOT-USleepNet, which divides multichannel EEG into temporal segments and constructs graph nodes from individual channel segments to model inter-channel relationships within the EEG modality. Chen et al. [19] proposed MSF-SleepNet, which further combines graph structure and a spatiotemporal attention mechanism to fuse multi-modal features. Ji et al. [20] proposed MixSleepNet, which uses convolution and frequency features to learn richer inter-modal relationships. Although these methods utilize the complementary effects between signals such as EEG and EOG, at a specific time point, only a few physiological signals often play a major role in determining sleep stages. Therefore, these methods have difficulty dynamically reflecting the importance of different physiological signals such as EEG and EOG in determining sleep stages based on the location and source of local key waveforms. The relevant methods and their limitations in characterizing local modality contributions are summarized in Table 2.

Sleep stages have continuous transition patterns, and the judgment of the sleep stage of the current epoch not only depends on its own signal characteristics, but also on the adjacent epoch states and stage transition relationships. The contextual information between epochs is also very helpful for sleep staging. Phan et al. [21] proposed SeqSleepNet, which models sleep staging as a sequence classification task and learns the stage transition relationship between consecutive epochs through an RNN. Seo et al. [22] proposed IITNet, which introduces a bidirectional LSTM after convolutional encoding to extract contextual features within and between epochs. Phan et al. [23] subsequently proposed L-SeqSleepNet to further extract long sequence information within the overnight range. Phan et al. [24] proposed XSleepNet, which extracts convolutional and frequency features as epoch-level features and inputs them into a dual-branch RNN to enhance the richness of features in context modeling. Phan et al. [25] proposed SleepTransformer, which uses a Transformer to model sequences of sleep stages in place of a recurrent architecture, thereby avoiding the vanishing gradient problem encountered by RNNs when processing long sequences. Bao et al. [26] proposed FFTCN, which uses a 1D-CNN and a 2D-CNN to extract features from raw EEG and continuous wavelet transform time–frequency representations, respectively. The compressed and fused epoch features are then fed into a TCN for classification. These methods demonstrate that cross-epoch context modeling can improve sleep staging performance. However, such methods learn more about the phase transition relationships at the epoch level, and local key waveforms may have been compressed or weakened before entering the context feature extraction process, making it difficult to fully utilize them in cross-epoch modeling. The relevant methods and their limitations in cross-epoch context modeling are summarized in Table 3.

**Figure 1:**
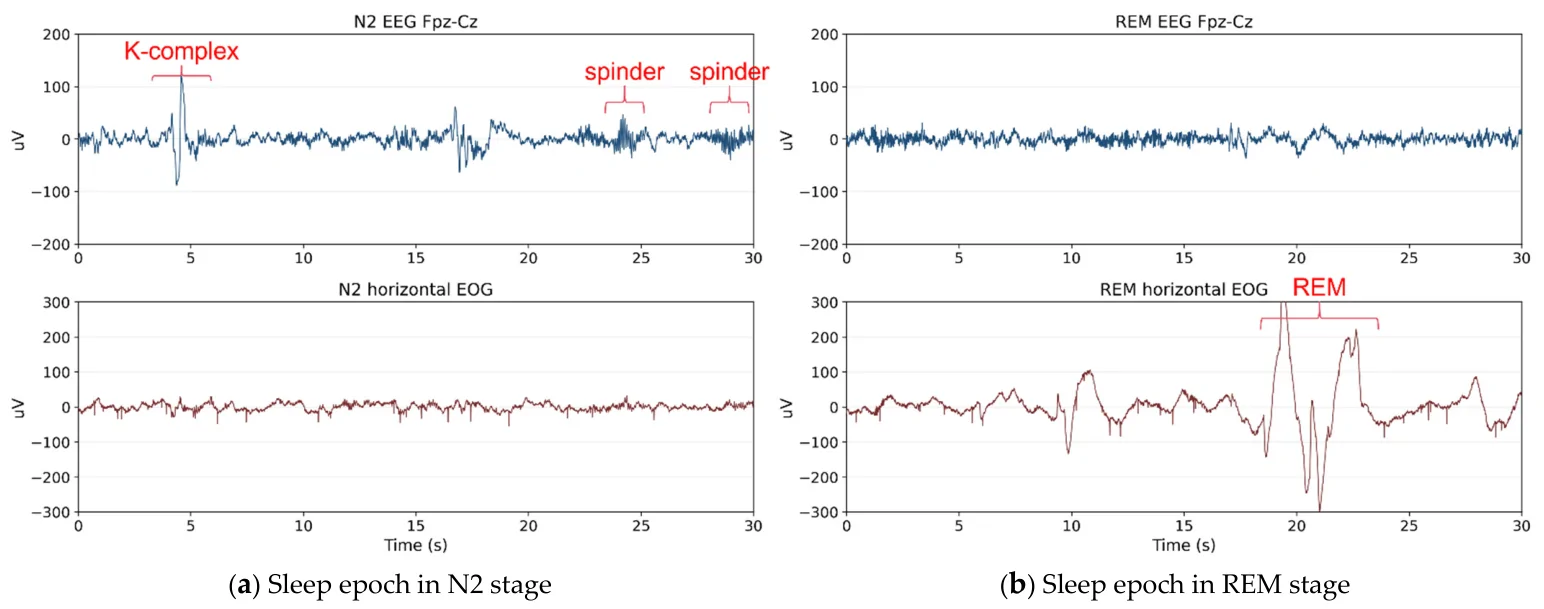
Two sleep signal segments.

Through summarizing the existing literature, we found the following shortcomings in existing research: (1) In most cases, there may be one or more key waveforms in a single epoch that are only a few seconds long. This waveform determines the sleep stage of the epoch. If feature extraction is performed on the entire epoch, key waveforms are easily obscured by non-key waveforms, weakening the distinguishability of the epoch. As shown in Figure 1a, the characteristic sleep spindle waves (waveforms with frequencies of 11–14 Hz) or k-complex waves (short-term waveforms that first decrease and then increase when the contextual waveform is flat) in the N2 stage are usually manifested as short-term waveforms. When one of these two types of waveforms appears in an epoch, it can be determined that the epoch belongs to the N2 stage. But the duration of these two types of waveforms is very short (usually 0.5–2 s), so they are easily obscured by other non-key waveforms. (2) At the same time point, only a few physiological signals play a dominant role in sleep staging, and existing methods have not evaluated the importance of different signals. As shown in Figure 1a, sleep spindle waves and k-complex waves appear in the EEG signal, but the eye movement waveform is relatively flat. As shown in Figure 1b, there is REM (rapid eye movement) in the EOG signal, but the EEG signal is relatively flat. (3) The sleep stage has transition patterns between sleep stages, and the staging of an epoch should also consider the key waveforms of the contextual epoch. Existing methods often downsample the feature vectors of each epoch to a very low dimension when extracting contextual information with temporal relationships, which results in the loss of key waveform features that can distinguish different sleep stages.

In order to make up for the shortcomings of existing research, this paper proposes MM-SleepNet, an automatic sleep staging network based on modal re-weighting and Mamba. MM-SleepNet divides epochs into smaller, overlapping patches and uses patches as the basic unit for feature extraction, signal weight adjustment, and extraction of epoch context information with temporal relationships. This partitioning method is helpful for capturing short-term key waveforms through patches, as well as representing the trend of longer waveforms (such as slow waves in the N3 period) through the distribution of continuous patches. The main contributions of this article are as follows:

(1) A patch-based PSG partitioning method was proposed, and a patch feature extractor was designed to extract waveform features from each patch, ultimately encoding each epoch as temporally ordered patch features.
(2) A dynamic modal re-weighting (DMR) module was designed. DMR calculates the importance of different signals across patches at the same time point and obtains patch features that capture differences in local modal contributions.
(3) A bidirectional Mamba encoder (BME) with positional encoding was designed to extract contextual features from adjacent epochs. Unlike traditional methods that directly calculate adjacent-epoch contextual features, we learn contextual information from patch features to preserve highly discriminative key waveform features.
(4) Comparative and ablation experiments were conducted on the Sleep-EDF and ISRUC datasets to validate the effectiveness of the proposed MM-SleepNet and its modules.

The rest of this paper is organized as follows: Section 2 defines the problem under study and provides a detailed introduction to the proposed MM-SleepNet. This is followed by comparative experimental results and ablation experimental results reported in Section 3. Finally, Section 4 concludes this paper.

## 2. Method

### 2.1. Problem Statement

Automatic sleep staging can be expressed as a mapping problem from continuous PSG physiological signals to sleep stage label sequences. For the overnight PSG recording of a subject, the continuous signal is first divided into *T* consecutive 30 s epochs. The epoch sequence is denoted as *X* = {*x*_1_,*x*_2_,⋯,*x_t_*,⋯,*x_T_*}, where *T* represents the total number of epochs included in a subject record. Among them, *x_t_* ∈ ℝ*^n^*^×*C*^, and *n* is the number of sampling points. The number of channels is *C* = *C^EEG^* + *C^EOG^*; *C^EEG^* represents the number of channels in EEG, and *C^EOG^* represents the number of channels in EOG. The sleep stage label sequence corresponding to the input sequence is denoted as *Y* = {*y*_1_,*y*_2_,⋯,*y_t_*,⋯,*y_T_*}, where *y_t_* represents the sleep stage of the *t*-th epoch. The goal of automatic sleep staging is to learn the mapping function *F*:*X* → *Y*, so that the model can predict the corresponding sleep stage based on continuous PSG signals.

### 2.2. Network Architecture of MM-SleepNet

**Figure 2:**
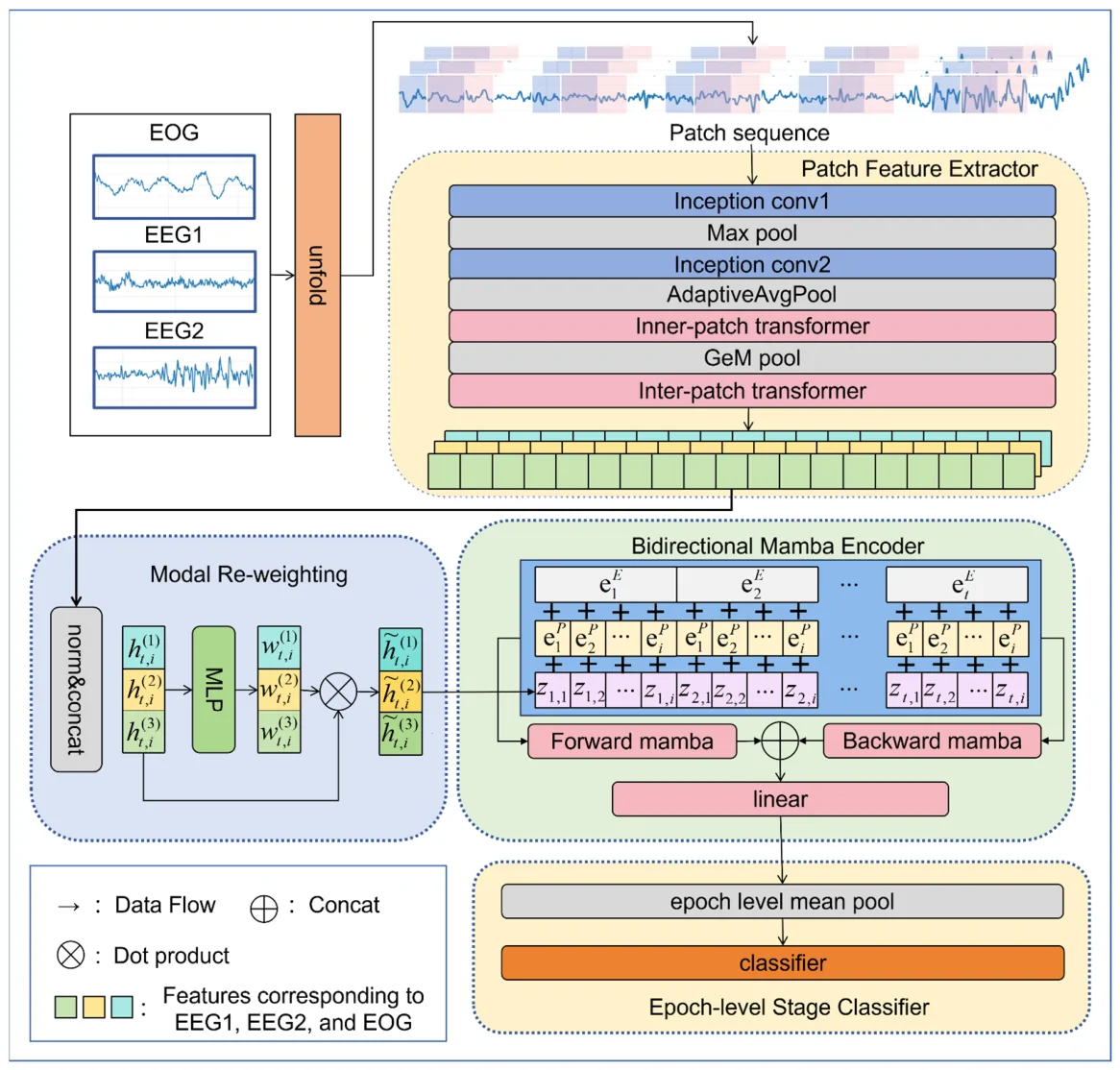
Network architecture of MM-SleepNet.

The architecture of the proposed MM-SleepNet is shown in Figure 2. EEG1 and EEG2 in Figure 2 come from different lead positions of EEG, which can record brain electrical activity from different spatial locations. Due to the possible differences in the performance of discriminative waveforms such as sleep spindle waves, K-complex, and slow waves on different leads, using two EEG inputs can help preserve more complete EEG discriminative information. EOG is used to provide eye movement information, which is helpful for distinguishing between the N1 and REM sleep stages.

MM-SleepNet performs overlapping patch partitioning on epochs from various inputs. The patch feature extractor (PFE) extracts features for each patch. The dynamic modal re-weighting (DMR) module evaluates the importance of the three input branches, EEG1, EEG2, and EOG, at the same time point, forming multi-modal local segment features that include modal importance. Afterward, MM-SleepNet unfolded the multi-modal patch features of consecutive epochs into a long sequence in chronological order and used bidirectional Mamba to learn the sleep stage transition relationships between adjacent epochs. Finally, the epoch sleep classifier aggregates features within the same epoch and outputs corresponding sleep stage prediction results.

### 2.3. Patch-Based PSG Partitioning Method

**Figure 3:**
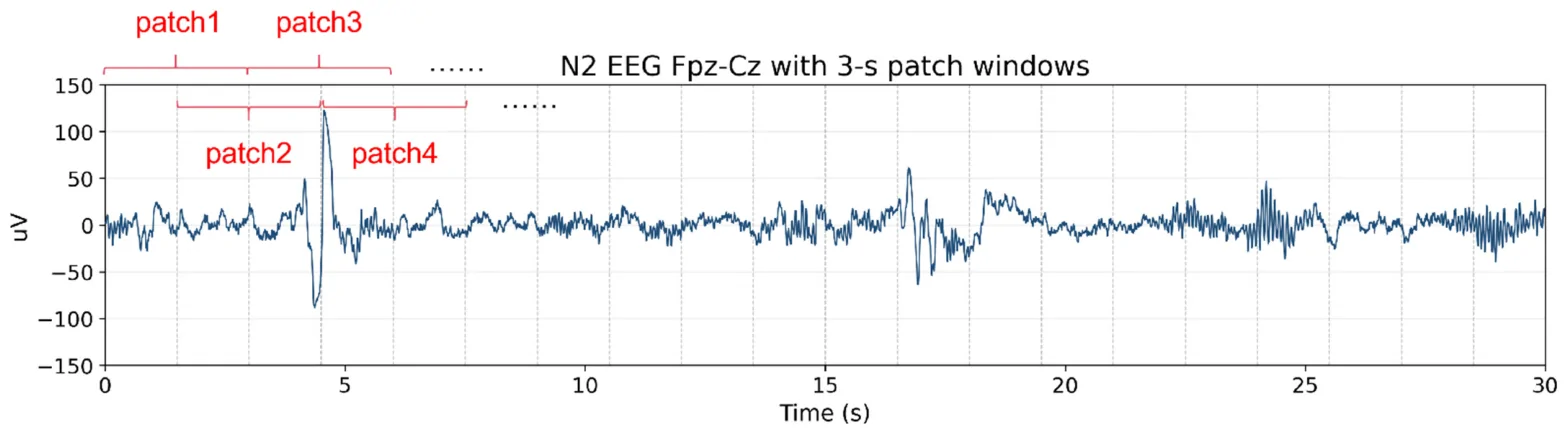
Schematic diagram of PSG divided into overlapping patches.

MM-SleepNet divides each epoch into time-ordered and partially overlapping patch sequences and only performs convolution operations within the patches. This can avoid mixing local key waveforms with background waveforms throughout the entire epoch during the feature extraction stage, thereby allowing key waveforms, such as sleep spindles, K-complexes, slow waves, and eye-movement-related changes, to form relatively independent patch-level representations. Figure 3 shows a schematic diagram of dividing PSG into overlapping patches. The length of sleep spindle waves and k-complex waves is 0.5–2 s.

The number of patches for each epoch is determined by the epoch length, patch length, and sliding step size. The formula for calculating the number of patches is as follows:

(1) $$P=\left\lfloor \frac{T_{\mathrm{epoch}}-T_{\mathrm{patch}}}{T_{\mathrm{stride}}}\right\rfloor +1$$

where *P* represents the number of patches in each epoch, *T_epoch_* represents the length of an epoch, *T_patch_* represents the length of the patch, and *T_stride_* represents the sliding step size, all of which are measured in seconds.

The epoch is 30 s, and setting the step size to half of the patch is beneficial for extracting the features of the patch more completely. There is a k-complex wave in Figure 3, which is partially divided into patch3 and partially divided into patch4. Setting the step size to half of the patch ensures that the waveform is fully feature extracted.

We found through experiments that the network performs best when the length of the patch is 3 s. Therefore, we set the length of each patch to 3 s, with a 1.5 s overlap between adjacent patches. The signal in Figure 3 is a real EEG signal. As shown in Figure 3, patch3 precisely contains the k-complex wave in the signal. Obviously, if feature extraction is performed on patches, more key waveform features will be preserved compared to feature extraction on epochs.

### 2.4. Patch Feature Extractor

**Figure 4:**
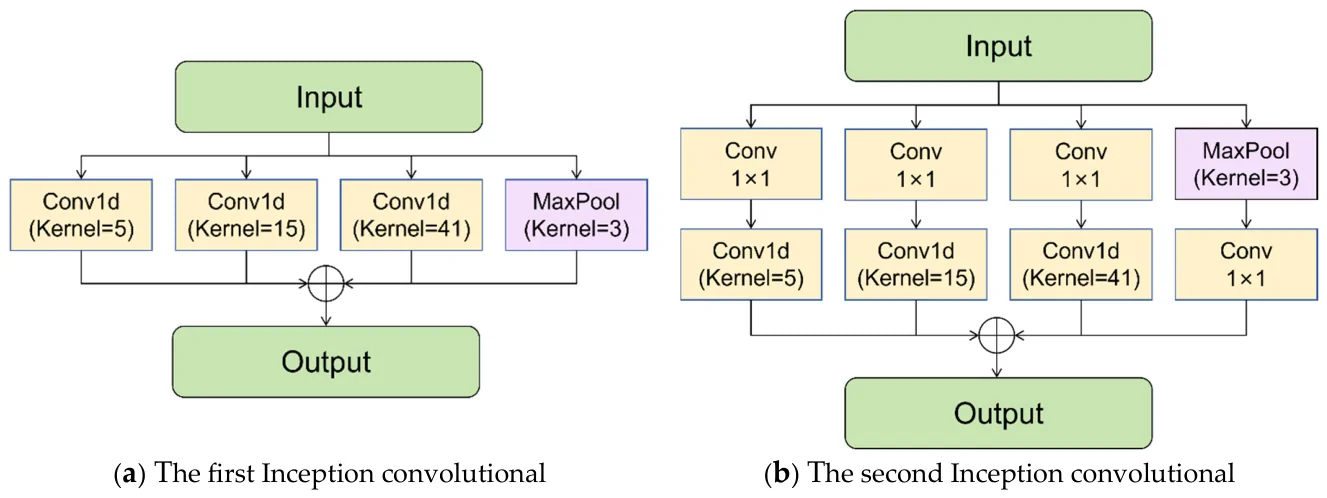
Structure of Inception convolution.

The box containing the patch feature extractor in Figure 2 illustrates the structure of the patch-feature extractor. The input of the patch feature extractor is a patch sequence. Inspired by Inception multi-scale convolution [27], we placed two Inception convolutions in the patch feature extractor. The structural of the first and second Inception convolutional are shown in Figure 4. The convolution kernel sizes of the three scales in the Inception convolutional structure are 5, 15, and 41, respectively. The kernel size of the Max pool in the Inception convolutional structure is 3. The kernel size of the Max pool layer between the two Inception convolutional structures in Figure 2 is 5. If we consider the two Inception convolutional structures and the middle Max pool layer as a whole, we can calculate the receptive field of these three layers using Formulas (2)–(4).

*r_l_* = *r_l_*_−1_ + (k − 1) × *S*_*l*−1_ (2)

Among them, *r_l_* represents the receptive field of the *l* layer, k represents the size of the convolution kernel, and *s_l_* represents the stride of the convolution in the *l* layer. The step size for both Inception convolution and Max pooling is 2. The calculation formula for the receptive field of the overall structure obtained from Formulas (3) and (4) is as follows:

*r*(k_1_,k_2_) = k_1_ + (5 − 1) × 2 + (k_2_ − 1) × 2 × 2 (3)

After simplification,

*r*(k_1_,k_2_) = k_1_ + 4k_2_ + 4 (4)

If the frequency of the signal is 100 Hz, substituting k_1_ = 3 and k_2_ = 3 (i.e., when the kernels in Figure 4a,b are both 3, the receptive field of the overall structure is minimized) into Formula (4), it can be seen that the minimum range of the receptive field of the above three layers is the sampling point within 0.19 s. By substituting k_1_ = 41 and k_2_ = 41 into Formula (4), it can be seen that the maximum range of the receptive field of the overall structure mentioned above is the sampling point within 2 s. The duration of sleep spindle waves and k-complex waves typically ranges from 0.5 s to 2 s. Therefore, two Inception convolutions can capture both the local details of a patch waveform with a small receptive field and the relatively complete trend of a key waveform with a large receptive field. The Max pool in Figure 4 can record the amplitude of the signal through downsampling.

**Figure 5:**
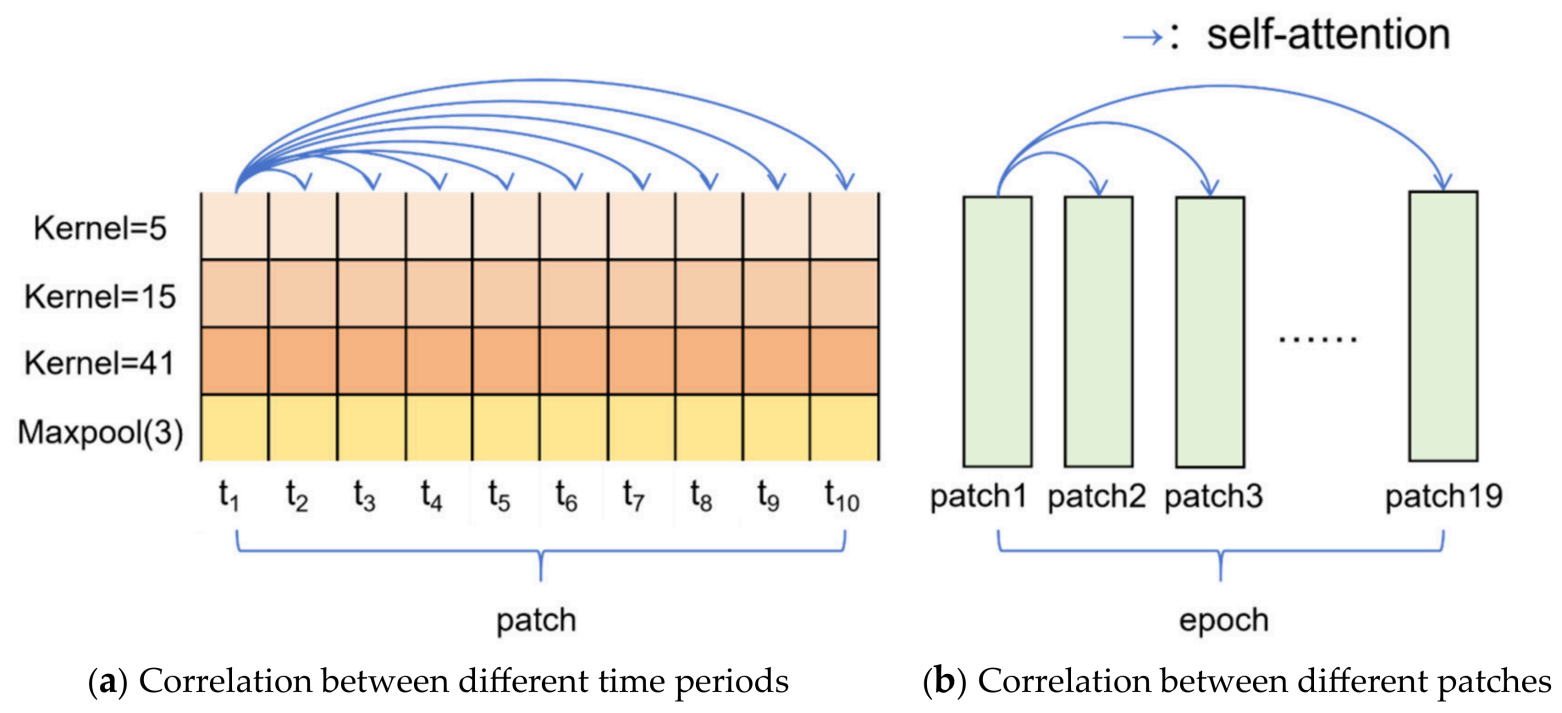
Schematic diagram of the calculation process of the inner patch transformer and inter-patch transformer.

The signal processed by the above structure is then compressed in the time dimension to 10 through the AdaptiveAvgPool layer. This parameter value was set to 10 based on experience, and we found that setting it to 10 was almost optimal in the experiment. However, small changes in this parameter value have little impact on the performance of the network. Afterward, the inner-patch transformer is used to calculate temporal feature correlations within each patch. As shown in Figure 5a, the transformer’s self-attention mechanism [28] is used to calculate the correlation between the features corresponding to each moment and those corresponding to other moments. Because the characteristic of k-complex waves is a short-term waveform that first decreases and then increases when the context waveform is flat, calculating feature correlation within the patch can capture the correlation between the decreasing and increasing waveforms to help determine the presence of k-complex waves. After the features are subjected to Generalized Mean Pooling (GeM pool) [29], a transformer is used to calculate the correlation between the features corresponding to different patches (Figure 5b). The function of the inter-patch transformer is to capture the difference between features containing k-complex waves and smooth contextual waveform features.

### 2.5. Dynamic Modal Re-Weighting Module

The structure of the dynamic modal re-weighting module is shown in the blue box in Figure 2. Let $h_{t,i}^{\left(m\right)}$ represent the feature of the i-th patch input on the *m* channel within the *t* epoch. Here, *m* ∈ {1,2,3} correspond to EEG1, EEG2, and EOG, respectively. Each $h_{t,i}^{\left(m\right)}$ is normalized within the patch. A multi-layer perceptron maps the concatenated features to generate a scoring matrix *a_t_*_,*i*_.

(5) $$a_{t,i}=\mathrm{MLP}\left(h_{t,i}^{\left(1\right)}⊕h_{t,i}^{\left(2\right)}⊕h_{t,i}^{\left(3\right)}\right)$$

In Formula (5), *MLP*(·) represents a three-layer fully connected multi-layer perceptron used for scoring, with an output dimension of 3, corresponding to three input branches: EEG1, EEG2, and EOG. ⊕ stands for feature concatenation. The scores are normalized with softmax and used as the dynamic weights for the current patch:

(6) $$\omega_{t,i}=\mathrm{softmax}(a_{t,i})\;=\;[\omega_{t,i}^{\left(1\right)},\omega_{t,i}^{\left(2\right)},\omega_{t,i}^{\left(3\right)}]$$

In Formula (6), $\omega_{t,i}^{\left(m\right)}$ represents the weight of the input branch of the *m* path at the position of the *t* epoch and the *i* patch. Multiply each feature $h_{t,i}^{\left(m\right)}$ by its corresponding weight $\omega_{t,i}^{\left(m\right)}$ to obtain the weighted features ${\tilde{h}}_{t,i}^{\left(1\right)}$, ${\tilde{h}}_{t,i}^{\left(2\right)}$, and ${\tilde{h}}_{t,i}^{\left(3\right)}$ in the blue box of Figure 2.

### 2.6. Bidirectional Mamba Encoder Considering Positional Encoding

There is a certain continuous evolution pattern between adjacent epochs of sleep signals. Existing methods typically compress each epoch when learning contextual information from adjacent epochs, making it difficult to further characterize the location of key waveforms. Especially in the N1 stage, the sample size is relatively small, and it is often adjacent to stages such as Wake and N2 in the overnight sleep sequence. Its waveform characteristics have certain similarities with adjacent stages, making it more prone to confusion with adjacent stages. To address this issue, we have designed a bidirectional Mamba encoder with positional encoding (BME). The schematic diagram of the BME structure is shown in the green box in Figure 2. BME arranges the patch features in consecutive epochs in the order of their respective epochs to form a patch sequence that preserves the key waveform positions. A bidirectional Mamba network [30] is used to extract the contextual relationships between epochs in both forward and backward temporal directions. BME first unfolds the epoch patch hierarchical structure into a one-dimensional sequence and then uses Formula (7) to calculate the positional encoding of patches and epochs [28].

(7) eposSk=sinpos10,000k/D,if k isevencospos10,000k−1/D,otherwise

Among them, S ∈{*E*,*P*} represents whether to use this formula to calculate the positional encoding of epoch or patch, pos represents the position of epoch or patch in the sequence, k is the feature index, and D is the feature dimension. Formula (8) adds positional encoding to the input features of BME.

(8) $${\tilde{z}}_{t,i}=z_{t,i}+e_{t}^{E}+e_{i}^{P}$$

Among them, $z_{t,i}\;=\;\left[{\tilde{h}}_{t,i}^{\left(1\right)}\;⊕\;{\tilde{h}}_{t,i}^{\left(2\right)}\;⊕\;{\tilde{h}}_{t,i}^{\left(3\right)}\right]$ and $e_{t}^{E}$ represent the positional encoding of the *t*-th epoch, $e_{i}^{P}$ represents the positional encoding of the *i*-th patch, and ${\tilde{z}}_{t,i}$ represents the patch feature that includes epoch and patch positional encodings.

**Figure 6:**
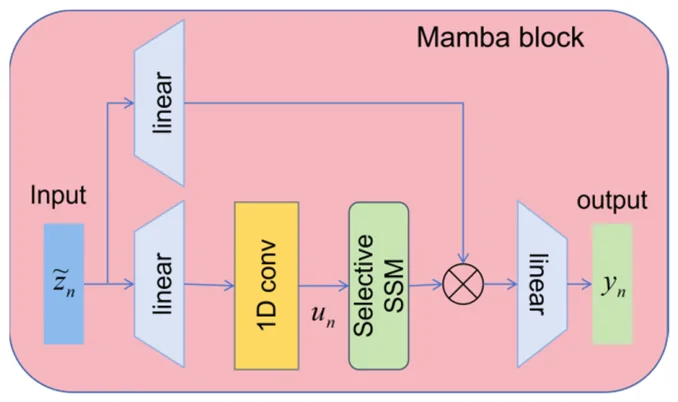
Structure of the Mamba block.

The structure of the Mamba block is shown in Figure 6. The feature sequence added with positional encoding and unfolded in chronological order is denoted as is denoted by $\tilde{z}=[{\tilde{z}}_{1},\ldots ,{\tilde{z}}_{N}]$, where *N* is the sequence length, and ${\tilde{z}}_{n}$ corresponds to the feature ${\tilde{z}}_{t,i}$ at the same position defined above. This sequence serves as the input to the bidirectional Mamba encoder. Projections and local convolution within the block produce the features *u_n_* fed into the SSM module.

The formula for recursive calculation of SSM on patch sequences is as follows:

*n* = *P* × (*t* − 1) + *i* (9)

(10) $$h_{n}={\bar{A}}_{n}h_{n-1}+{\bar{B}}_{n}u_{n}$$

*y_n_* = *C_n_h_n_* + *D_skip_u_n_* (11)

Here, *P* is the number of patches within each epoch. For a 30 s epoch, a window length of 3 s and a stride of 1.5 s yield *P* = 19. *h_n_*_−1_ represents the hidden state at the previous position. It propagates historical information through the discretized state transition matrix ${\bar{A}}_{n}$. The current input features *u_n_* are mapped into the hidden state space by the input mapping matrix ${\bar{B}}_{n}$ and added to the historical information to obtain the hidden state *h_n_* at the current position. Subsequently, *h_n_* is mapped by the output matrix *C_n_* and added to the direct feedthrough term *D_skip_u_n_* to obtain the SSM output *y_n_* at the current position.

The state update parameters vary with the input, allowing the model to adjust the retention of historical information and the incorporation of current information according to the sequence content. The SSM output *y_n_* then passes through gating and output projection within the block to produce the output of the Mamba branch.

On this basis, the bidirectional Mamba block uses a bidirectional scanning method to enhance its contextual modeling capability:

(12) $$Y_{f}=\mathrm{Mamba}(\tilde{z})$$

(13) $$Y_{b}=\mathrm{Reverse}(\mathrm{Mamba}(\mathrm{Reverse}(\tilde{z})))$$

Among them, *Y_f_* represents the forward output obtained by scanning from the starting point to the ending point of the sequence, and *Y_b_* represents the reverse output obtained by reversing the sequence and then scanning and restoring the order. The outputs from both directions are concatenated along the feature dimension and mapped back to the input dimension through a linear layer.

This study uses Mamba-1 with a model dimension of 384 in each branch. The state dimension, local convolution width, and expansion factor are set to 16, 4, and 2, respectively. The dropout probability is set to 0.3.

### 2.7. Classifier and Loss Function

The classifier consists of an average pooling layer, 3 fully connected layers, and Softmax. MM-SleepNet uses cross-entropy loss function to train the network.

(14) $$L_{\mathrm{CE}}=-\frac{1}{T}\sum_{t=1}^{T}\sum_{c=1}^{C}y_{t,c}\log\left(p\right)$$

Among them, *y_t_*_,*c*_ ∈ {0, 1} represents the true label of the *t*-th epoch for class *c*, and *p* represents the probability that the network assigns to that class.

## 3. Experiment

### 3.1. Dataset

To verify the effectiveness of the proposed MM-SleepNet in automatic sleep staging tasks, we conducted a series of experiments on the Sleep Cassette subset of the Sleep-EDF dataset [31], the Subgroup 1 subset, and the Subgroup 3 subset of the ISRUC dataset [32]. These datasets are commonly used in sleep staging network assessment. Table 4 summarizes the basic information of the three datasets.

The Sleep Cassette subset of the Sleep-EDF dataset contains 153 overnight PSG records from 78 healthy subjects, totaling 195,479 sleep epochs. The original record contains 2 EEG signals and 1 EOG signal. Fpz-Cz, Pz-Oz, and horizontal EOG were selected as inputs in the experiment. The original labels of this dataset were labeled according to the R&K [33] standard for each epoch, including Wake, N1, N2, N3, N4, REM, MOVEMENT, and UNKNOWN. To unify the experiment into a five-class classification task, we merged N3 and N4 into N3 according to AASM standards [1,34], and removed the MOVEMENT and UNKNOWN labels. Considering that overnight recordings typically contain longer awake segments, the experiment only retained the effective sleep interval from 30 min before the first non-wake phase to 30 min after the last non-wake phase.

The Subgroup 1 subset of the ISRUC dataset contains overnight PSG records of 100 adult sleep disorder subjects, while the Subgroup 3 subset contains overnight PSG records of 10 healthy adult subjects. All records were labeled as Wake, N1, N2, N3, and REM by sleep experts according to AASM standards [1,34]. Each PSG contains 6 EEG channels (C4-A1, O2-A1, F4-A1, C3-A2, O1-A2, and F3-A2) and 2 EOG channels (LOC-A2 and ROC-A1). Each epoch lasts for 30 s with a sampling rate of 200 Hz. To maintain input consistency, this article resamples the dataset to 100 Hz.

### 3.2. Experimental Design

In this paper, the proposed MM-SleepNet is implemented based on the PyTorch 2.1.2 framework [35] and trained in an end-to-end manner. For the Sleep-EDF dataset, the network input consists of two EEG channels and one EOG channel, with Fpz-Cz and Pz-Oz as EEG1 and EEG2 inputs, respectively, and horizontal EOG as the EOG input. For the ISRUC dataset, the network also uses two EEG inputs and one EOG input. The EEG1 branch inputs are C4-A1, O2-A1, and F4-A1; the EEG2 branch inputs are C3-A2, O1-A2, and F3-A2; and the EOG branch inputs are LOC-A2 and ROC-A1. Except for the input lead configuration, the two datasets adopt the same training strategy. During the training process, the Adam optimizer [36] is used, with a maximum number of training epochs of 100, batch size of 16, initial learning rate of 1 × 10^−4^, and weight decay coefficient of 1 × 10^−4^. Evaluate performance once on the validation set after each round of training. This paper adopts an early-stopping mechanism with the goal of maximizing validation metrics. After 25 rounds of training, early stopping is enabled with a patience value of 15. Set the number of data loading threads to 4. The network training adopts the cross-entropy loss function.

Tenfold cross-validation divided by subjects is used as the evaluation strategy. For the Sleep-EDF dataset and ISRUC dataset, we divided the training set, validation set, and test set in an 8:1:1 ratio based on the subjects. The above measures ensure that the sleep segments of the same subject will not appear simultaneously during the training and testing phases. This setting can more accurately evaluate the generalization ability of the network among different subjects and is consistent with common protocols in existing sleep staging studies.

This paper uses Accuracy (ACC), Macro-F1 (MF1), Cohen’s kappa (*κ*), and F1 score for each sleep category as evaluation indicators. ACC is used to measure the overall classification accuracy. MF1 is the average value of the F1 score for each category, which is more suitable for observing the overall performance under imbalanced categories. Cohen’s kappa is used to evaluate the consistency between predicted labels and true labels. This paper uses average sensitivity (Sens) and average specificity (Spec) as supplementary evaluations.

If *TP*, *TN*, *FP*, and *FN* are the number of true positive, true negative, false positive, and false negative samples, respectively, then the formula for calculating *ACC* is as follows:

(15) $$\mathrm{ACC}=\frac{\mathrm{TP}+\mathrm{TN}}{\mathrm{TP}+\mathrm{TN}+\mathrm{FP}+\mathrm{FN}}$$

The *F*1 score of the *i* class is denoted as *F*1*_i_* and is used to analyze the classification performance of the model at Wake, *N*1, *N*2, *N*3, and REM stages. Its calculation formula is as follows:

(16) $${F1}_{i}=\frac{2{\mathrm{TP}}_{i}}{2{\mathrm{TP}}_{i}+{\mathrm{FP}}_{i}+{\mathrm{FN}}_{i}}$$

*MF*1 is the average of all categories’ *F*1 scores, calculated using the following formula:

(17) $$\mathrm{MF}1=\frac{1}{N}\sum_{i=1}^{N}{F1}_{i}$$

Denote *M* as the total number of sleep epochs, *n_ij_* as the number of epochs with real label *i* and predicted label *j*, ${n_{i}}^{\mathrm{true}}$ as the number of epochs with real label *i*, and ${n_{i}}^{\mathrm{pred}}$ as the number of epochs with predicted label *i*. The observed consistency rate *p_o_* and random consistency rate *p_e_* are calculated as follows:

(18) $$p_{o}=\frac{1}{M}\sum_{i=1}^{N}n_{\mathrm{ii}}$$

(19) $$p_{e}=\frac{1}{M^{2}}\sum_{i=1}^{N}{n_{i}}^{\mathrm{true}}{n_{i}}^{\mathrm{pred}}$$

The formula for Cohen’s kappa (denoted by *κ*) calculation is as follows:

(20) $$\kappa =\frac{p_{o}-p_{e}}{1-p_{e}}$$

where *p_o_* represents the observed consistency rate between predicted and true labels, while *p_e_* represents the expected consistency rate under random conditions.

The calculation formulas for Sens and Spec are as follows:

(21) $$\mathrm{Sens}=\frac{1}{N}\sum_{i=1}^{N}\frac{{\mathrm{TP}}_{i}}{{\mathrm{TP}}_{i}+{\mathrm{FN}}_{i}}$$

(22) $$\mathrm{Spec}=\frac{1}{N}\sum_{i=1}^{N}\frac{{\mathrm{TN}}_{i}}{{\mathrm{TN}}_{i}+{\mathrm{FP}}_{i}}$$

In Formulas (15)–(22), *N* represents the number of categories, and in this paper, *N* = 5.

### 3.3. Comparison Results

We selected eight methods, XSleepNet [24], MMASleepNet [14], SleePyCo [7], CareSleepNet [9], TSEDSleepNet [10], ST-USleepNet [17], MSF-SleepNet [19], and MixSleepNet [20], for comparison with the proposed MM-SleepNet.

Table 5, Table 6 and Table 7 present the comparative experimental results of various methods on the Sleep-EDF dataset and the Subgroup 1 and Subgroup 3 subsets of the ISRUC dataset, respectively.

As can be seen from Table 5, the evaluation indicators ACC, MF1, and κ of MM SleepNet exceeded the baseline TSEDSleepNet by 0.6, 1.1, and 0.6, respectively, but the improvement was not significant. This is a limitation of this method. However, the N3 period contains very little data, making it difficult to classify. The F1 score of MM-SleepNet exceeds the strongest baseline by 3.4%. Improving the accuracy of difficult classification samples may be more helpful for diagnosing certain diseases, which may be the significance of our method. Although N3 has discrimination criteria such as slow wave activity, it only accounts for 6.5% in this dataset, and the model is prone to insufficient learning from it. MM-SleepNet achieved the best results in N1 and N3 stages, indicating that our network can fully utilize local discriminative waveforms and continuous information from adjacent epochs in minority classes.

Unlike the Sleep-EDF dataset, which mainly includes healthy subjects, ISRUC-S1 comprises subjects with sleep disorders, and the two datasets differ in sleep-stage distributions. As can be seen from Table 6, the ACC and kappa of MM-SleepNet improved by 0.1% and 0.2%, respectively, compared with the second-best method. The MF1 of MM-SleepNet was similar to that of CareSleepNet and only 0.1% lower. MM-SleepNet achieved the best F1 scores for Wake, N1, N2, and REM, with only the N3 score lower than that of MSF-SleepNet. It should be noted that MSF-SleepNet uses more physiological modalities, including EEG, EOG, EMG, and ECG, whereas MM-SleepNet uses only EEG and EOG. This study focuses on sleep staging with the EEG and EOG signals that are commonly used and readily available in clinical practice. The additional physiological modalities may provide supplementary discriminative information, which may explain why MSF-SleepNet performs better than MM-SleepNet for N3. In addition, compared with MSF-SleepNet, MM-SleepNet improved the overall ACC, MF1, and kappa by 0.7%, 0.8%, and 0.9%, respectively. Compared with ST-USleepNet, the ACC increased by 0.8%. Therefore, although MM-SleepNet did not achieve the best result for N3, it still achieved good overall performance with fewer input modalities.

On the ISRUC-S3 dataset, although the proposed MM-SleepNet achieved the best overall performance, with ACC, MF1, and kappa improving by 0.6%, 0.7%, and 0.8%, respectively, compared to suboptimal methods, the magnitude of the improvement was not significant. The Wake F1 score was 0.6% lower than that of MSF-SleepNet, owing to the additional performance provided by its use of more physiological modalities. MM-SleepNet achieved the best F1 scores for N1, N2, N3, and REM, indicating that it retained strong overall discriminative ability on the healthy-population data from ISRUC-S3 with fewer input modalities.

Based on the experimental results on three datasets, it can be seen that the proposed MM-SleepNet outperforms other models in overall metrics and performs well in N1, which has a low proportion and is prone to confusion. Compared with models that use more modalities, MM-SleepNet does not rely on additional physiological signal stacking to improve performance but instead improves feature utilization efficiency through patch-level local evidence extraction, patch-wise importance adjustment, and cross-epoch bidirectional context modeling.

### 3.4. Confusion Matrices and Hypnogram of Prediction

**Figure 7:**
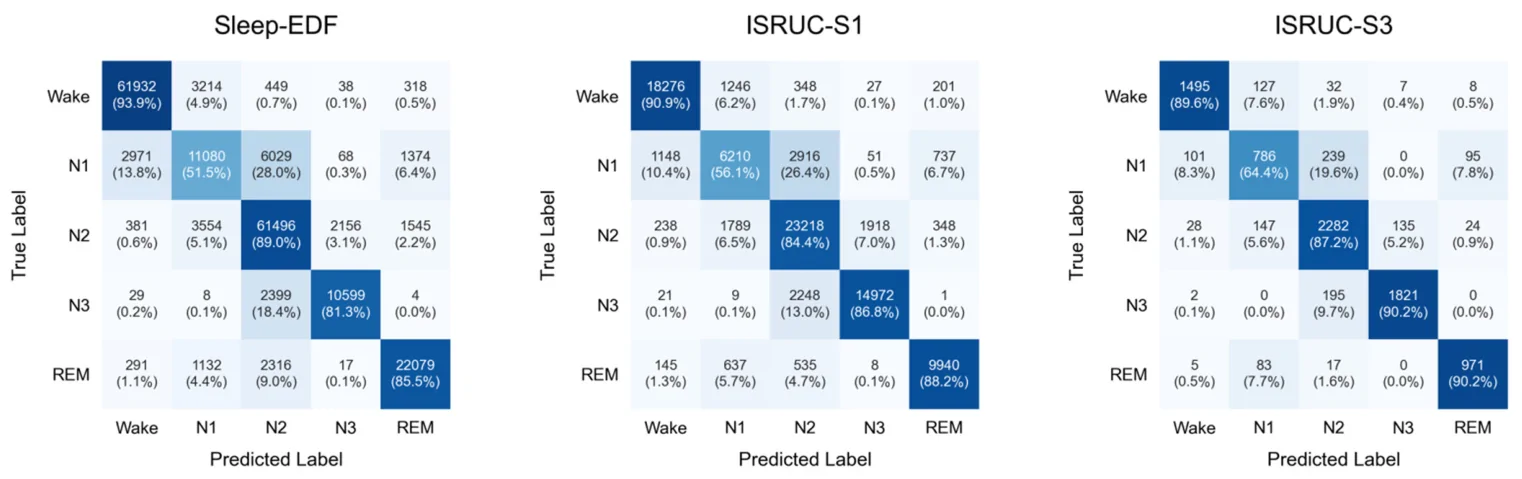
The confusion matrix of MM-SleepNet on the Sleep-EDF, ISRUC-S1, and ISRUC-S3 datasets.

Figure 7 shows the confusion matrix of MM-SleepNet on the Sleep-EDF, ISRUC-S1, and ISRUC-S3 datasets. The confusion matrix reveals the confusion situation of MM-SleepNet in distinguishing different sleep stages. From Figure 7, it can be seen that the overall prediction results on the three datasets show a high proportion of diagonal lines, indicating that our model can achieve stable sleep stage discrimination on most samples. Among them, the four categories of Wake, N2, N3, and REM have high accuracy on all three datasets. Compared to other categories, N1 is still the most difficult stage to identify, with accuracy rates of 51.5%, 56.1%, and 64.4% on the Sleep-EDF, ISRUC-S1, and ISRUC-S3 datasets, respectively. This is because N1 is located in the boundary area of the transition from wakefulness to stable sleep, and its EEG and eye movement characteristics are not typical enough, making it easy to confuse with Wake, N2, and even REM. The main error of the model in this paper is concentrated between N1 and N2, and there is also a small amount of confusion between REM and N1 or N2. The error in the N3 stage is mainly manifested as being misclassified as N2. This indicates that the model has been able to distinguish stable sleep stages well, but there are still shortcomings in the transition stage samples.

**Figure 8:**
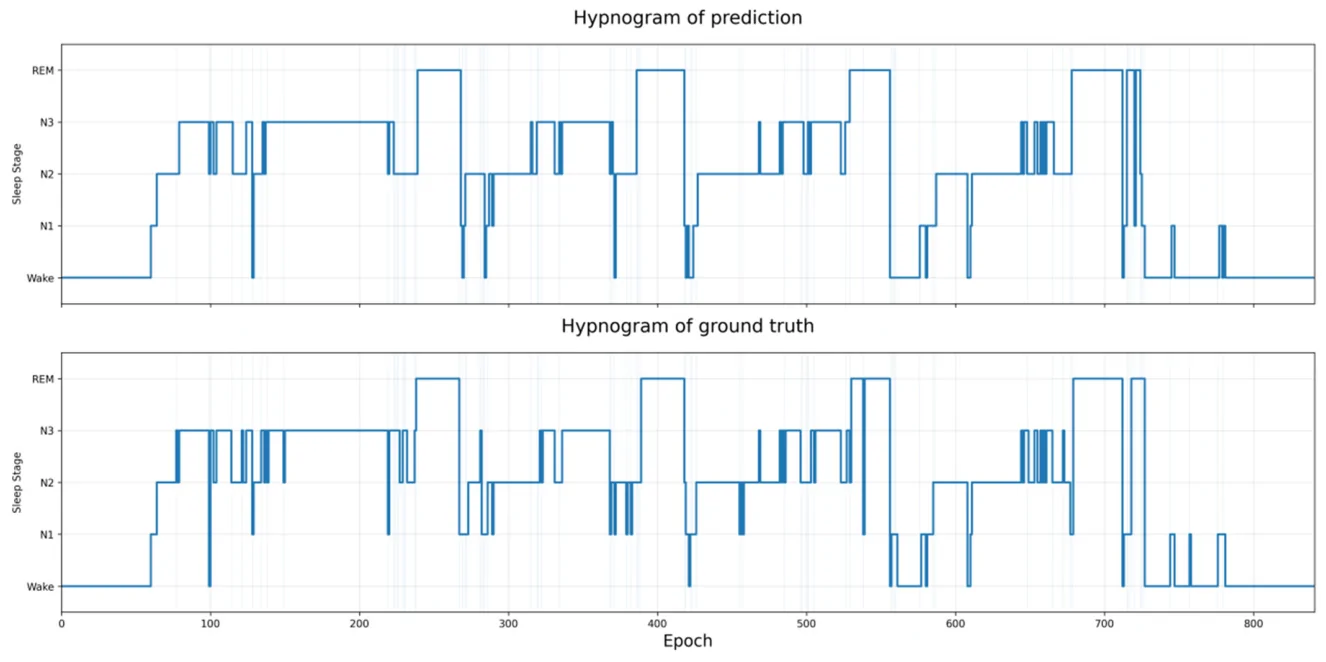
Hypnogram of prediction and ground truth.

Figure 8 shows the ground truth and prediction results of a volunteer’s overnight sleep record in the Sleep-EDF dataset. MM-SleepNet can accurately track the overall changes in the sleep structure throughout the night, including the transition from light sleep to deep sleep after falling asleep, the emergence of periodic REM stages, and frequent switching between late-night sleep stages. Most sleep segments with longer duration and more stable structure can be correctly predicted, indicating that our model not only has discriminative ability at the single epoch level, but also maintains temporal consistency over a longer period of time.

MM-SleepNet shows stability in both category differentiation and overnight structure maintenance. The confusion matrix indicates that the model has strong recognition ability for stages such as Wake, N2, N3, and REM. The overnight example demonstrates that the model can preserve the overall pattern of sleep stages changing over time. The patch-level local fragment modeling of PFE, patch-wise re-weighting of DMR, and bidirectional temporal modeling of BME can balance local discriminability and overnight temporal consistency.

### 3.5. Patch Length Experiment

Table 8 shows the experimental results using different scales of patches on the Sleep-EDF dataset. Due to the step size being half the window length, a 4 s patch corresponds to a 2 s step size, a 3 s patch corresponds to a 1.5 s step size, and a 2 s patch corresponds to a 1 s step size. Therefore, the 2 s patch, 3 s patch, and 4 s patch generate 14, 19, and 29 patches, respectively.

The 3 s patch achieved the best results in all indicators, indicating that this length can better balance local waveform preservation and contextual expression. Compared to the 2 s patch, the 3 s patch contains more complete sleep spindle waves, K-complexes, and eye-movement-related segments, which helps the model extract stable discriminative features. Compared to 4 s patches, 3 s patches can reduce the mixing of irrelevant background signals in long segments and avoid diluting local key waveforms. Therefore, using 3 s as the patch length is more reasonable.

### 3.6. Sensitivity and Specificity

We evaluated the sensitivity and specificity of MM-SleepNet on the Sleep-EDF dataset and compared it with XSleepNet, MMASleepNet, and SlePyCo. The two indicators are calculated according to Formulas (21) and (22), and the results are shown in Table 9.

The Sens and Spec of MM-SlepNet are 80.22% and 96.12%, respectively, which are higher than the three comparison methods.

### 3.7. Model Complexity Analysis

To further analyze the relationship between model performance and computational overhead, we compared the parameter count and floating-point computation count FLOP of multi-modal sleep grading methods with similar overall performance during inference. We conducted this experiment on the Sleep-EDF dataset. The results are shown in Table 10.

According to Table 10, our model has 6.19 M parameters, and the FLOPs are approximately 9.84 G. Compared with TSEDSleepNet, which has 11.3 M parameters, our model uses approximately 45.2% fewer parameters. Compared with CareSleepNet, which has 19.6 M parameters, our model uses approximately 68.4% fewer parameters, and its FLOPs are lower. TSEDSleepNet and CareSleepNet both use EEG and EOG signals, so they were selected as comparison methods for complexity. Compared with the Transformer used by these methods, the Mamba used in MM-SleepNet can effectively model temporal and contextual relationships with fewer parameters. The Inception convolutional structure also has relatively low computational cost.

Based on the experimental results in Table 6 and Table 7, it can be seen that our model outperforms other models in overall performance. Even if the best performance is not achieved on individual datasets or category metrics, it can still achieve better or closer performance than existing methods at lower parameter and computational costs. This result indicates that our model not only maintains good classification performance but also has certain advantages in controlling model complexity.

### 3.8. Module Ablation Experiment

To analyze the contributions of each module of MM-SleepNet, this paper constructed different network variants on the Sleep-EDF dataset for ablation experiments, and the results are shown in Table 11. Among them, PFE represents retaining only the patch feature extractor of MM-SleepNet, BME represents adding bidirectional Mamba encoder with positional encoding to the model, DMR represents adding dynamic modal re-weighting module to the model, and TF represents using a two-layer Transformer to replace the sequence modeling structure in BME.

According to Table 11, when using PFE alone, the model achieved 83.5% ACC, 77.2% MF1, and 76.3% kappa, indicating that patch-level epoch encoding can provide effective local fragment evidence for sleep staging. After adding BME on this basis, ACC increased to 85.5%, MF1 increased to 79.8%, and kappa increased to 79.7%, with a significant overall performance improvement. This indicates that unfolding the patches within the epoch in chronological order and further conducting bidirectional context modeling is the main source of performance improvement for the model in this paper. In the third row of Table 11, when DMR is added to PFE alone, the overall indicator changes are not significant, indicating that only performing patch-wise gating and re-weighting is not sufficient to stably improve the final classification performance. However, when DMR was combined with BME, the ACC, MF1, and kappa of the complete model reached 85.8%, 80.8%, and 80.3%, respectively. Among them, MF1 increased by 1.0% compared to PFE+BME, and F1 score increased from 51.0% to 54.7% in the N1 stage. This indicates that DMR is more suitable for use with subsequent context modeling modules. DMR first adjusts the contribution of different patch local segments, and BME then uses these re-weighted patch sequences to learn the continuous changes between adjacent epochs, thereby improving the recognition performance in confusing stages. Compared with PFE+DMR+TF, the complete model achieved better results in ACC, MF1, and kappa, especially in the N1 stage where F1 score increased from 46.9% to 54.7%. This result indicates that on the epoch patch unfolding sequence constructed in this paper, BME is more suitable than two-layer Transformer for capturing long-range contextual dependencies. From the comprehensive ablation results, it can be seen that PFE, DMR, and BME, respectively, undertake the roles of local fragment evidence extraction, weight adjustment of signals at the same time, and cross-epoch context modeling. The combined effect of the three achieves the improvement of model performance.

### 3.9. Contribution and Weight Allocation for Different Patches

**Figure 9:**
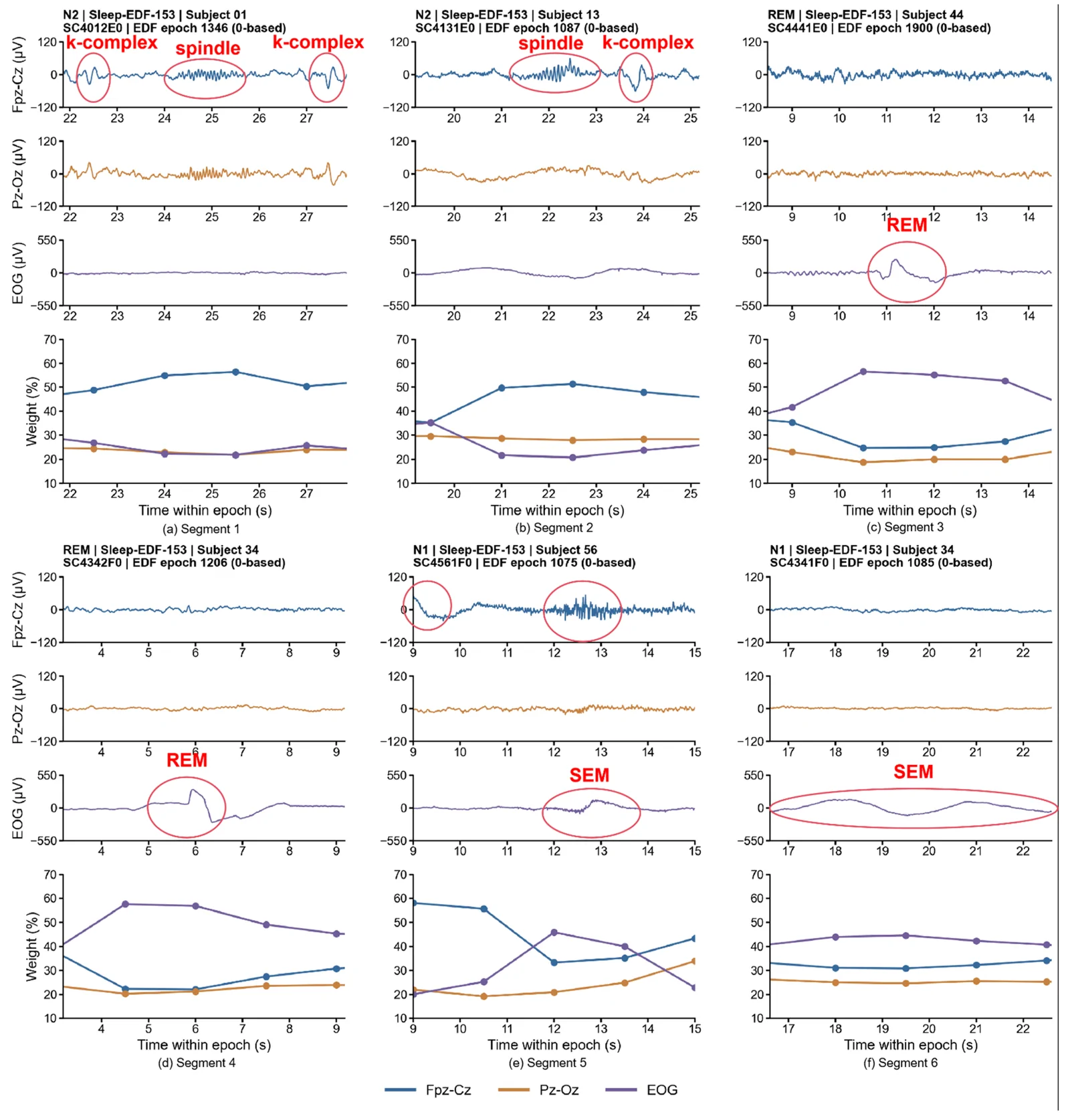
Visualization of local waveforms and modal weight allocation at different sleep stages.

We selected representative sleep signal segments from the dataset and demonstrated the different weights assigned by the DMR module based on the contributions of different patches at the same time. Figure 9 shows the allocation of local waveforms and modal weights during different sleep stages. The information in the upper left corner of each subgraph is as follows: sleep stage, dataset name, subject number, original record number, and epoch number. The epoch number starts counting from 0, starting from the first epoch of the original PSG. The subgraphs from top to bottom are Fpz-Cz, Pz-Oz, EOG signal, and corresponding modal weights. The horizontal axis represents the time within the epoch. The red circle indicates the position of the waveform being discussed. Spindle represents sleep spindle waves, k-complex represents K-complex waves, REM represents rapid eye movement, and SEM represents slow eye movement. The Fpz-Cz channel in Figure 9a exhibits K-complex waves around 22 s and 27 s, and spindle waves around 25 s, indicating that this channel consistently receives the highest weight within the display area. The weights of the three channels on the left side of Figure 9b are relatively close. As the patch covers the spindle waves around 22 s, Fpz-Cz obtains the highest weight and maintains this advantage in the K-complex region around 24 s.

The EOG channels in Figure 9c,d exhibit distinct REM waves around 11 s and 6 s, respectively, and the patches covering these activities are assigned the highest weights to EOG. After moving away from these activities, the weight gap between EOG and other channels gradually narrows.

The Fpz-Cz waveform on the left side of Figure 9e shows significant changes, indicating that this channel has obtained the highest weight. However, when SEM waves appear around 12 s to 13 s, even if Fpz-Cz still exhibits significant oscillations during the same time period, the highest weight still shifts to EOG. In Figure 9f, the EOG channel exhibits sustained slow eye movement and consistently obtains the highest weight within the display area. These cases demonstrate that the DMR module of MM-SleepNet is capable of obtaining features related to key waveforms during sleep stages and assigning higher weights to relevant channels in corresponding patches, with an allocation trend consistent with the displayed waveform features.

### 3.10. Modal Ablation Experiment

To analyze the impact of different input modalities on classification performance, we conducted modal ablation experiments on the Sleep-EDF dataset and compared the results using only EEG, only EOG, and both EEG and EOG. The experimental results are shown in Table 12.

According to Table 12, the EEG+EOG bimodal input achieved the best overall results, with ACC, MF1, and kappa higher than those obtained with EEG or EOG alone. This result indicates that the simultaneous use of EEG and EOG can utilize the complementary information between the two physiological signals, thereby improving the performance of the sleep staging network.

Comparing the results of the two single-modal models, it can be found that the model has strong discriminative ability when only EEG is used, and the overall performance is significantly higher than when only EOG is used, indicating that EEG is still the main source of information in sleep staging. Especially in the N2 and N3 stages, the F1 scores obtained using EEG reached 86.3% and 81.6%, respectively, significantly higher than those obtained using EOG alone. This is consistent with the characteristic that N2 and N3 mainly rely on EEG features such as EEG rhythm, sleep spindle waves, K-complexes, and slow wave activity for discrimination. On the other hand, although EOG has relatively weak overall performance, it achieved an 85.4% F1 score in the REM stage, indicating that eye-movement-related changes have auxiliary discriminative value for sleep staging tasks.

On this basis, after integrating EEG and EOG, the performance of MM-SleepNet was further improved in the Wake, N1, and REM stages. Among them, N1 increased from 51.3% to 54.7%, and REM increased from 83.6% to 86.3%. This indicates that EEG can provide a stable basis for discrimination, while EOG can supplement eye-movement-related information for stages such as N1 and REM. The DMR in MM-SleepNet re-weights the contributions of different modalities at the patch level, allowing the model to adaptively utilize the complementary features of EEG and EOG based on local segment content.

## 4. Conclusions

This paper proposes a network for sleep staging: MM-SleepNet. MM-SleepNet divides each epoch into several overlapping patches, alleviating the problem of key waveforms being obscured by non-key waveforms. Afterward, MM-SleepNet calculates the importance of different signals on patches at the same time point through a dynamic re-weighting module. Finally, MM-SleepNet uses BME to learn contextual information across epochs to adapt to the transition patterns between sleep stages. To verify the effectiveness of MM-SleepNet, we conducted a series of experiments on the Sleep Cassette subset of the Sleep-EDF dataset, the Subgroup 1 subset, and the Subgroup 3 subset of the ISRUC dataset. The experimental results show that the proposed MM-SleepNet has improved on all indicators compared to the comparison methods, but the improvement is not significant. However, MM-SleepNet has lower computational complexity and better performance for difficult classification. The results of the ablation experiment demonstrate the effectiveness of various parts of MM-SleepNet.

## Figures and Tables

**Table 1:** Studies related to key waveform feature extraction.

Authors	Methodology	Limitation
Eldele et al. (2021) [6]	MRCNN + AFR + TCE AttnSleep	Downsampling before recalibration may weaken key waveforms (Sleep-EDF-78 N1 F1: 42.1%).
Lee et al. (2024) [7]	CNN + SCL + Transformer SleePyCo	Epoch encoding may weaken waveform location and detail (Sleep-EDF N1 F1: 50.4%).
Wan et al. (2025) [8]	DWT + CNN + BiLSTM + SEAM	The artificial statistical features have limited representation of key waveforms. (Sleep-EDF-20 N1 F1: 53.56%).
Wang et al. (2024) [9]	CNN + Transformer CareSleepNet	Convolutions may mix key and background waveforms, with high computational cost (19.6 M parameters, 11.7 GFLOPs).
Zhang et al. (2024) [10]	CNN + Transformer TSEDSleepNet	Downsampling may weaken waveform details, with a large parameter count (11.3 M).
Huang et al. (2025) [11]	MSCNN + Mamba + SE + CapsNet EEGCMCNet	Convolution and pooling may weaken key waveform details (Sleep-EDF-78 N1 F1: 41.1%).

**Table 2:** Studies related to local modality contributions.

Authors	Methodology	Limitation
Jia et al. (2021) [13]	U^2^-Net + MSE + MMA SalientSleepNet	Additive fusion does not explicitly distinguish local modality contributions (Sleep-EDF-153 ACC: 84.1%).
Zheng et al. (2022) [14]	MBFE + AFF (SE + TE) MMASleepNet	Waveform-specific modality contributions are insufficiently characterized (Sleep-EDF-153 ACC: 82.6%).
Hu et al. (2025) [15]	MSCNN + Transformer + Cross-modal attention block	Local modality contributions are not explicitly characterized (SingleSleepPlus, ISRUC-S3 N1 F1: 53.37%).
Dai et al. (2023) [16]	STFT + Transformer MultiChannelSleepNet	Local modality weights are not explicitly distinguished (Sleep-EDF-78 REM F1: 82.6%).
Ma et al. (2025) [17]	GCN + U-Net ST-USleepNet	Modality weights at key waveform locations are not explicitly distinguished (ISRUC-S3 ACC: 82.93%).
Liu and Zhang (2025) [18]	STOT + GCN + U-Net + CBAM	Only single mode is considered. Weak REM features remain difficult to distinguish (ISRUC-S3 REM F1: 81.8%).
Chen et al. (2025) [19]	GCN + GRU + CL MSF-SleepNet	Waveform-specific modality contributions are insufficiently characterized (ISRUC-S3 MF1: 83.8%).
Ji et al. (2024) [20]	3D-CNN + GCN MixSleepNet	Local modality contributions are not explicitly distinguished (ISRUC-S3 N1 F1: 62.5%).

**Table 3:** Studies related to feature compression in cross-epoch context modeling.

Authors	Methodology	Limitation
Seo et al. (2020) [22]	ResNet + BiLSTM IITNet	Introduces sub-epoch encoding across epochs, but N1 performance remains limited (Sleep-EDF-20 N1 F1: 43.4%).
Phan et al. (2023) [23]	Hierarchical BiLSTM L-SeqSleepNet	Epoch compression limits cross-epoch transfer of key waveform information (Sleep-EDF-20 N1 F1: 45.3%).
Phan et al. (2022) [24]	CNN + RNN XSleepNet	Context modeling uses compressed epoch-level time–frequency features (Sleep-EDF-78 ACC: 84.0%).
Phan et al. (2022) [25]	Transformer SleepTransformer	Epoch compression limits cross-epoch transfer of key waveform information (Sleep-EDF-78 ACC: 81.4%).
Bao et al. (2024) [26]	CWT + 1D-CNN + 2D-CNN + TCN FFTCN	Compression before TCN limits cross-epoch transfer of key waveform information (ISRUC-S1 N1 F1: 44.98%).

**Table 4:** Basic information of the datasets used in experiments.

Dataset	Subjects	Samples	Wake	N1	N2	N3	REM	Scoring	k-Fold
Sleep-EDF	78	195,479	33.7	11.0	35.4	6.7	13.2	R&K	10-fold
ISRUC-S1	100	87,187	23.1	12.7	31.6	19.8	12.9	AASM	10-fold
ISRUC-S3	10	8600	19.4	14.2	30.4	23.5	12.5	AASM	10-fold

**Table 5:** Comparative experimental results on the Sleep-EDF dataset.

Method		Overall Metrics			Per-Class F1 Score				
System	Model	ACC	MF1	κ	Wake	N1	N2	N3	REM
XSleepnet [24] (2021)	EEG, EOG	84.0	77.9	77.8	-	-	-	-	-
MMASleepNet [14] (2022)	EEG, EOG, EMG	82.6	77.6	76.1	92.8	49.0	84.9	81.2	79.7
SleePyCo [7] (2024)	EEG	84.6	79	78	93.5	50.4	86.5	80.5	84.2
CareSleepNet [9] (2024)	EEG, EOG	85.1	80.4	78.9	-	-	-	-	-
TSEDSleepNet [10] (2025)	EEG, EOG	85.2	79.7	79.7	93.6	54.6	86.8	78.4	85.5
Our Model	EEG, EOG	85.8	80.8	80.3	94.5	54.7	86.7	81.8	86.3

**Table 6:** Comparative experimental results on the ISRUC-S1 dataset.

Method		Overall Metrics			Per-Class F1 Score				
System	Model	ACC	MF1	κ	Wake	N1	N2	N3	REM
MMASleepNet [14] (2022)	EEG, EOG, EMG	79.0	77.5	73.0	87.8	54.0	77.0	85.3	83.3
CareSleepNet [9] (2024)	EEG, EOG	83.2	81.8	78.1	-	-	-	-	-
MixSleepNet [20] (2024)	EEG, EOG, EMG ECG	81.3	78.7	75.7	90.8	51.2	79.9	87.1	84.4
ST-USleepNet [17] (2025)	EEG, EOG, EMG ECG	82.5	81.8	-	-	-	-	-	-
MSF-SleepNet [19] (2025)	EEG, EOG, EMG ECG	82.6	80.9	77.4	91.2	57.0	81.2	88.4	86.5
Our Model	EEG, EOG	83.3	81.7	78.3	91.5	59.3	81.8	87.5	88.4

**Table 7:** Comparative experimental results on the ISRUC-S3 dataset.

Method		Overall Metrics			Per-Class F1 Score				
System	Model	ACC	MF1	κ	Wake	N1	N2	N3	REM
MMASleepNet [14] (2022)	EEG, EOG, EMG	81.9	80.6	76.7	88.8	59.5	82.0	87.3	86.8
MixSleepNet [20] (2024)	EEG, EOG, EMG ECG	83.0	82.1	78.2	89.9	62.5	81.9	89.9	86.0
ST-USleepNet [17] (2025)	EEG, EOG, EMG ECG	82.9	82.4	-	-	-	-	-	-
MSF-SleepNet [19] (2025)	EEG, EOG, EMG ECG	84.9	83.8	80.5	91.2	64.5	83.7	91.0	88.8
Our Model	EEG, EOG	85.5	84.5	81.3	90.6	66.5	84.8	91.5	89.3

**Table 8:** Effect of patch length on model performance.

Input	Overall Metrics			Per-Class F1 Score				
	ACC	MF1	κ	Wake	N1	N2	N3	REM
2 s patch	85.6	80.4	80.0	93.9	52.9	86.4	81.6	86.0
3 s patch	85.8	80.8	80.3	94.5	54.7	86.7	81.8	86.3
4 s patch	85.5	79.8	79.6	93.8	51.4	86.5	81.6	86.1

**Table 9:** Sens and Spec of different methods.

Method	Sens (%)	Spec (%)
XSleepNet [24]	77.60	95.70
MMASleepNet [14]	77.61	95.45
SleePyCo [7]	78.00	95.86
Our Model	80.22	96.12

**Table 10:** Comparison of model complexity.

Method	Input	# Param	FLOPs
TSEDSleepNet	EEG, EOG	11.3 M	-
CareSleepNet	EEG, EOG	19.6 M	11.7 G
Our Model	EEG, EOG	6.19 M	9.84 G

**Table 11:** Module ablation experiment results.

System	Overall Metrics			Per-Class F1 Score				
	ACC	MF1	κ	Wake	N1	N2	N3	REM
PFE	83.5	77.2	76.3	93.7	44.6	85.2	82.4	79.8
PFE+BME	85.5	79.8	79.7	93.9	51.0	86.9	81.1	86.4
PFE+DMR	83.6	77.1	77.0	93.7	44.2	85.2	82.1	80.3
PFE+DMR+TF	84.7	78.6	78.7	93.8	46.9	85.8	82.0	84.9
MM-SleepNet	85.8	80.8	80.3	94.5	54.7	86.7	81.8	86.3

**Table 12:** Modal ablation experimental results.

Input	Overall Metrics			Per-Class F1 Score				
	ACC	MF1	κ	Wake	N1	N2	N3	REM
EEG	84.9	79.4	78.9	94.0	51.3	86.3	81.6	83.6
EOG	81.6	74.6	74.3	91.4	42.6	82.9	70.6	85.4
EEG+EOG	85.8	80.8	80.3	94.5	54.7	86.7	81.8	86.3

## Data Availability

This study used the publicly available Sleep Cassette subset of the Sleep-EDF Database Expanded [31] and Subgroups 1 and 3 of the publicly available ISRUC-Sleep dataset [32]. The datasets are available at https://physionet.org/content/sleep-edfx/1.0.0/ and https://sleeptight.isr.uc.pt/ (accessed on 7 September 2026), respectively.

## References

[1] Iber C., Ancoli-Israel S., Chesson A.L., Quan S.F. (2007). The AASM Manual for the Scoring of Sleep and Associated Events: Rules, Terminology and Technical Specifications.

[2] Zhang R., Jiang R., Hu H., Gao Y., Xia W., Song B. (2025). Automatic Sleep Staging Method Using EEG Based on STFT and Residual Network. IEEE Access.

[3] Fraiwan L., Lweesy K., Khasawneh N., Wenz H., Dickhaus H. (2012). Automated sleep stage identification system based on time-frequency analysis of a single EEG channel and random forest classifier. Comput. Methods Programs Biomed..

[4] Alickovic E., Subasi A. (2018). Ensemble SVM method for automatic sleep stage classification. IEEE Trans. Instrum. Meas..

[5] Supratak A., Dong H., Wu C., Guo Y. (2017). DeepSleepNet: A model for automatic sleep stage scoring based on raw single-channel EEG. IEEE Trans. Neural Syst. Rehabil. Eng..

[6] Eldele E., Chen Z., Liu C., Wu M., Kwoh C.K., Li X., Guan C. (2021). An attention-based deep learning approach for sleep stage classification with single-channel EEG. IEEE Trans. Neural Syst. Rehabil. Eng..

[7] Lee S., Yu Y., Back S., Seo H., Lee K. (2024). SleePyCo: Automatic sleep scoring with feature pyramid and contrastive learning. Expert Syst. Appl..

[8] Wan Q., Bo Y., Zhang Y., Li M., Wang X., Chen C., Liu L., Wu W. (2025). Automated sleep staging from single-channel electroencephalogram using hybrid neural network with manual features and attention. iScience.

[9] Wang J., Zhao S., Jiang H., Zhou Y., Yu Z., Li T., Li S., Pan G. (2024). CareSleepNet: A hybrid deep learning network for automatic sleep staging. IEEE J. Biomed. Health Inform..

[10] Zhang Z., Lin B.-S., Peng C.-W., Lin B.-S. (2024). Multi-modal sleep stage classification with two-stream encoder-decoder. IEEE Trans. Neural Syst. Rehabil. Eng..

[11] Huang L., Zhang W., Li C., Zhao W., Peng H., Chen X. (2025). EEGCMCNet: A hybrid network for sleep-stage classification. IEEE Sens. J..

[12] Chambon S., Galtier M.N., Arnal P.J., Wainrib G., Gramfort A. (2018). A deep learning architecture for temporal sleep stage classification using multivariate and multimodal time series. IEEE Trans. Neural Syst. Rehabil. Eng..

[13] Jia Z., Lin Y., Wang J., Wang X., Xie P., Zhang Y. (2021). SalientSleepNet: Multimodal salient wave detection network for sleep staging. Proceedings of the International Joint Conference on Artificial Intelligence.

[14] Zheng Y., Luo Y., Zou B., Zhang L., Li L. (2022). MMASleepNet: A multimodal attention network based on electrophysiological signals for automatic sleep staging. Front. Neurosci..

[15] Hu Y., Yang X., Xu Y., Sun J. (2025). Causal-aware reliability assessment of single-channel EEG for transformer-based sleep staging. Front. Neurosci..

[16] Dai Y., Li X., Liang S., Wang L., Duan Q., Yang H., Zhang C., Chen X., Li L., Li X. (2023). MultiChannelSleepNet: A transformer-based model for automatic sleep stage classification with PSG. IEEE J. Biomed. Health Inform..

[17] Ma J., Lin Q., Jia Z., Feng M. (2025). ST-USleepNet: A spatial-temporal coupling prominence network for multi-channel sleep staging. Proceedings of the Thirty-Fourth International Joint Conference on Artificial Intelligence.

[18] Liu L., Zhang C. (2025). STOT-USleepNet: A study of sleep stage classification based on EEG signals. Proceedings of the 2025 International Conference on Big Data and Intelligent Medicine (BDIMed).

[19] Chen J., Fan X., Ge R., Xiao J., Wang R., Ma W., Li Y. (2025). Towards interpretable sleep stage classification with a multi-stream fusion network. BMC Med. Inform. Decis. Mak..

[20] Ji X., Li Y., Wen P., Barua P., Acharya U.R. (2024). MixSleepNet: A multi-type convolution combined sleep stage classification model. Comput. Methods Programs Biomed..

[21] Phan H., Andreotti F., Cooray N., Chen O.Y., De Vos M. (2019). SeqSleepNet: End-to-end hierarchical recurrent neural network for sequence-to-sequence automatic sleep staging. IEEE Trans. Neural Syst. Rehabil. Eng..

[22] Seo H., Back S., Lee S., Park D., Kim T., Lee K. (2020). Intra- and inter-epoch temporal context network (IITNet) using sub-epoch features for automatic sleep scoring on raw single-channel EEG. Biomed. Signal Process. Control.

[23] Phan H., Le K.P., Huynh E., Chen O.Y., Tran M.C., Koch P., Mertins A., Baumert M., Mikkelsen K.B., De Vos M. (2023). L-SeqSleepNet: Whole-cycle long sequence modeling for automatic sleep staging. IEEE J. Biomed. Health Inform..

[24] Phan H., Chen O.Y., Tran M.C., Koch P., Mertins A., De Vos M. (2022). XSleepNet: Multi-view sequential model for automatic sleep staging. IEEE Trans. Pattern Anal. Mach. Intell..

[25] Phan H., Mikkelsen K., Chen O.Y., Koch P., Mertins A., De Vos M. (2022). SleepTransformer: Automatic sleep staging with interpretability and uncertainty quantification. IEEE Trans. Biomed. Eng..

[26] Bao J., Wang G., Wang T., Wu N., Hu S., Lee W.H., Lo S.L., Yan X., Zheng Y., Wang G. (2024). A feature fusion model based on temporal convolutional network for automatic sleep staging using single-channel EEG. IEEE J. Biomed. Health Inform..

[27] Szegedy C., Liu W., Jia Y., Sermanet P., Reed S., Anguelov D., Erhan D., Vanhoucke V., Rabinovich A. (2015). Going deeper with convolutions. Proceedings of the IEEE Conference on Computer Vision and Pattern Recognition.

[28] Vaswani A., Shazeer N., Parmar N., Uszkoreit J., Jones L., Gomez A.N., Kaiser Ł., Polosukhin I. (2017). Attention is all you need. Advances in Neural Information Processing Systems.

[29] Radenovic F., Tolias G., Chum O. (2018). Fine-tuning CNN image retrieval with no human annotation. IEEE Trans. Pattern Anal. Mach. Intell..

[30] Gu A., Dao T. (2024). Mamba: Linear-time sequence modeling with selective state spaces. Proceedings of the Conference on Language Modeling.

[31] Kemp B., Zwinderman A.H., Tuk B., Kamphuisen H.A.C., Oberye J.J.L. (2000). Analysis of a sleep-dependent neuronal feedback loop: The slow-wave microcontinuity of the EEG. IEEE Trans. Biomed. Eng..

[32] Khalighi S., Sousa T., Santos J.M., Nunes U. (2015). ISRUC-Sleep: A comprehensive public dataset for sleep researchers. Comput. Methods Programs Biomed..

[33] Rechtschaffen A., Kales A. (1968). A Manual of Standardized Terminology, Techniques and Scoring System for Sleep Stages of Human Subjects.

[34] Berry R.B., Brooks R., Gamaldo C.E., Harding S.M., Lloyd R.M., Quan S.F., Troester M.T., Vaughn B.V. (2017). AASM scoring manual updates for 2017 (version 2.4). J. Clin. Sleep Med..

[35] Paszke A., Gross S., Massa F., Lerer A., Bradbury J., Chanan G., Killeen T., Lin Z., Gimelshein N., Antiga L. (2019). PyTorch: An imperative style, high-performance deep learning library. Advances in Neural Information Processing Systems.

[36] Kingma D.P., Ba J. (2015). Adam: A method for stochastic optimization. Proceedings of the International Conference on Learning Representations.

